# Decentralisation of the Health System Derailed by Organisational Inertia in Machinga, Malawi

**DOI:** 10.34172/ijhpm.7956

**Published:** 2024-07-21

**Authors:** Sandram Erixy Naluso, MacDonald Isaac Kanyangale

**Affiliations:** ^1^Graduate School of Business and Leadership Studies, University of KwaZulu-Natal, Durban, South Africa.

**Keywords:** Decentralisation, Health System, Resistance, Inertia, Transition, Grounded Theory

## Abstract

**Background:**

Managing the transition of a health system (HS) from a centralised to a decentralised model has been touted as a panacea to the complex challenges in developing countries like Malawi. However, recent studies have demonstrated that decentralisation of the HS has had mixed effects in service provision with more dominant negative outcomes than positive results. The aim of this study was to develop a substantive grounded theory (GT) that elaborates on how activities of central decision-makers and local healthcare mangers shape the process of shifting the HS to a decentralised model in Machinga, Malawi.

**Methods:**

The study was qualitative in nature and employed the Straussian version of GT. Some participants were interviewed twice, and a total of 36 semi-structured interviews were conducted with 25 purposively selected participants using an interview guide. The interviews were conducted at the headquarters of the Ministry of Health (MoH) and other ministries and agencies, and in Machinga District. Data were analysed using open, axial, and selective coding processes of the GT methodology; and the conditional matrix and paradigm model were used as data analysis tools.

**Results:**

The findings of this study revealed seven different activities, forming two opposing and interactional sub-processes of enabling and impeding patterns that derailed the decentralisation drive. The study generated a GT labelled "decentralisation of the HS derailed by organisational inertia," which elaborates that decentralisation of the HS produced mixed results with more predominant negative outcomes than positive effects due to resistance at the upper organisational echelons and members of the District Health Management Team (DHMT).

**Conclusion:**

This article concludes that organisational inertia at the personal and strategic levels of leadership entrusted with decentralising the HS in Malawi, contributed immensely to the derailment of shifting the HS from the centralised to the decentralised model of health service provision.

## Background

**Key Messages**

**Implications for policy makers**

- This qualitative study has contributed a grounded theory (GT) elaborating how the process of decentralising the health system (HS) yielded mixed results in Malawi, and providing insights that policy-makers may identify with, avoid or consider avoiding.
- The study may guide policy-makers on how to manage a transition process and its complexity in Malawi or any similar developing country.
- The study may be of use to future researchers as it brings processual and multi-level approaches to the fore in understanding the emergent transition.
- The study is a catalyst for future researchers to extend their research on decentralisation to other disciplines, for example, education to explore if the transition to a decentralised model of service provision in any district will bring similar or different results.

**Implications for the public**

As decentralisation of the health system (HS) unfolds, structural and psychological changes affect people and the public at large. Notably, studies on the decentralisation of the HS focus on the structural aspects of change, which are external, and ignore the internal psychological transition of people. This study is beneficial to the public because it illuminates the naivety of focusing primarily on structural inertia to understand the HS, as this excludes the inner psychological dimension of decentralisation.

Globally, several studies on decentralisation of the health system (HS) have explored the potential benefits of decentralisation, including its effects on efficiency in service delivery.^1-4^ For example, Panda and Thakur^5^ examined the effects of decentralisation of the HS using macro-level indicators to improve management processes and health outcomes. Bossert,^6^ Liwanag and Wyss,^7^ and Suma and Baatiema^8^ employed the decision-space theory to examine the extent to which managerial decision-making is granted to local decision-makers to improve service delivery. Similar studies on the decision space in Pakistan, Bolivia, and Chile,^9^ India,^10^ Fiji,^11^ Philippines,^7,12^, Tanzania,^13^ Ghana, Malawi, and Uganda^14^ also examined the degree of authority that local decision-makers have over HS functions. Notably, these studies highlight freedom from central control and clinical empowerment as some of the benefits of decentralisation.^15,16^ Other studies have focused on the failed promises of decentralisation to improve efficiency and effectiveness in service delivery.^15,17^ However, one of the weaknesses of these studies is that they have not developed a grounded theory (GT) to elaborate on how the HS transitioned to a decentralised model in different health contexts.

 In developing countries, studies on decentralisation of the HS have predominantly focused on outcomes or results rather than the process of decentralising the HS. For example, Alonso-Garbayo et al^18^ in Uganda, Bossert^6^ in Fiji, Panda, and Thakur^5^ in India, studied decentralisation of the HS using case studies to understand results of the interventions in these countries. In India, Seshadri et al^10^ used a survey, while Inkoom and Gyapong^19^ in Ghana, Malawi, and Tanzania used literature review to establish the extent of decentralisation in these countries. Similarly, Roman et al^20^ in South Africa, and Dwicaksono and Fox^21^ in America, Asia, and Africa used systematic review, while Zaidi et al^22^ in Pakistan used document review. These studies predominantly focused on results and/or outcomes rather than the process of decentralisation.There is a dearth of GT studies elaborating on how the HS transitions to a decentralised model. While we know much about the use of methodologies such as case studes and surveys to unravel decentralisation and get a snapshot and quantitative view, little is known about the processual, situated and multi-dimensional perspective of decentralisation as a process. In this regard, existing studies have failed to capture temporal dynamics and social interactions to understand conditions under which decentralisation has been implemented.

 Although decentralisation is regarded as a panacea for improving service delivery in developing countries since the 1980s,^3,23,18^ its role on HS improvement is still limited and inconclusive.^21^ Available evidence suggests that achieving the theoretical promises of improving service delivery in most developing countries has been a big challenge.^3^ These challenges often stem from flaws in the implementation process.^24^ Some scholars attribute the challenges to an acute shortage of input resources, for example, health financing and medicines.^25^ As Mweninguwe^26^ asserts, “Nowadays, Malawi government’s health facilities are in a crisis, and some patients cannot even afford simple drugs like paracetamol. Parents are asked to buy from private clinics…” (p. 1). Other scholars attribute this to the narrow decision-space accorded to local healthcare managers, for example, District Health Management Team (DHMT) members.^14,18^ Notably, DHMTs not only need more power and authority to make decisions, but also more control over resources to be able to implement these decisions.^14^ Wiesche et al^27^ acknowledge that scholars have ignored the task of developing inductive theories to look at decentralisation as a process unfolding over time and its results.

 The objective of this qualitative study was to develop a substantive GT elaborating on the transition of a HS from a centralised to a decentralised model in Malawi. The article hinges on the research question: How did the activities of central decision-makers and healthcare managers shape the process of shifting the HS to a decentralised model in Machinga, Malawi? This study is significant in three ways: First, it elaborates on the complex process of managing the transition of a HS to a decentralised model of service provision. The theory developed in this study is valuable because it illuminates patterns of activities that shape (*enable* or *impede*) decentralisation of the HS. Second, the study is significant to various members of the DHMT as it brings to the fore realities in managing various resources. Lastly, the study is valuable to academicians as it illustrates the pragmatic use of the GT methodology to delve into the lived experiences of members of the DHMT and central decision-makers. The developed GT offers a theoretical basis for researchers to integrate process, HS, and managing complexity in future research and similar contexts, whether in Malawi or any other developing countries.

 Mindful that decentralisation is a process, not an event,^12^ there are four widely-accepted dimensions in management circles, and these are *administrative*, *political*, *fiscal*,and *market* decentralisation.^2,28,29^ The above dimensions are further developed into four typologies, namely: *deconcentration, devolution*, *delegation*, and *privatisation.*^28,30^ Bossert^31^ argues that the main form of decentralisation of HSs in developing countries is deconcentration. However, with reference to Malawi, Chiweza^32^ argues that the country adopted devolution as a form of decentralisation to replace deconcentration in line with the Local Government Act.^33^ This article begins by unravelling concepts of decentralisation and HS before presenting methods and findings. Lastly, there is a discussion of the findings and implications of the study.

## Methods

###  Context of the Study and Study Design 

 This qualitative study was part of a larger GT research of the transition of a HS from a centralised to a decentralised model in Machinga, Malawi. Originally, decentralisation was designed for implementation in phases using an incremental approach^34^ spanning for a period of 10 years from 2004 to 2014. However, its implementation dragged beyond 2014 due to the slow pace at which activities and functions were rolled-out to the district. This extended the period of implementation to more than 20 years at the time of data collection and analysis in 2022. Some activities and processes were implemented by central decision-makers to decentralise the HS to the district. For instance, decision-making powers over planning and policy formulation. Others were implemented by local healthcare managers (DHMT members) after decentralisation took-off. For example, financial allocation and supervision.

 Machinga District was chosen for the study because of two reasons: First, no study has been conducted in the district to generate a theory that elaborates on the process of decentralising the HS. Second, few studies conducted in Malawi have focused on results/outcomes and challenges of decentralising the HS rather than the entire process. Hence, there is a compelling need to conduct the study in the district focusing on process as this integrates means, outcomes and the context which are key to develop a substantive GT.

 The study adopted a constructivist paradigm to gain multiple and subjective realities on the transition of a HS.^35^ A Straussian version of GT was employed as an overarching methodology because of its simplicity in developing a substantive GT as exemplified by the systematic and procedural steps it takes to generate a theory.^36^ This version is more structured and gives more guidance than the other versions.^37^ The researcher subscribes to the constructivist paradigm because it involves social construction of meaning from the lived experiences of actors.^38^ In this regard, the study involves the researcher’s construction of meaning in liaison with members of the DHMT and central decision-makers to understand the process of decentralising the HS to Machinga.

 A critical incident technique was used as an interview method for investigating significant events or incidents identified by participants. Chell and Pittaway^39^ are cognizant of the merits of using the critical incident technique in qualitative research by asserting that it enhances the completeness of data.

###  Sampling

 The study used two sampling techniques, namely, purposive sampling and theoretical sampling. These techniques were ideal for the study because they support in generating a theory grounded in data. Purposive sampling was used to identify the first participant with experience in decentralising Malawi’s HS. On the other hand, theoretical sampling was used to select incidents relevant to the evolving theory.^35^ For example, the incidents labelled “resistance” and “inertia” were selected based on the emergent theory.

###  Inclusion and Exclusion Criteria 

####  Inclusion Criteria

 The selection criteria for the study had two aspects. First, only those individuals in managerial positions at the district or headquarters and with hands-on experience in managing the transition of a HS were selected as participants. The researcher selected the initial participant who could articulate and provide the most appropriate data about the phenomenon under study. After purposive sampling, subsequent selection of participants was done through theoretical sampling, which aimed at selecting incidents relevant to the evolving theory.^35^ Subsequently, this involved gathering incidents logically based on earlier data and the researcher’s analytical thinking.^40^ This implies that any subsequent data collection was guided by the previously collected data and the researcher’s knowledge. The second criterion was that participants should be those who could succinctly articulate their experience of decentralising the HS in Machinga District.

####  Exclusion Criteria

 Any prospective participant without experience and who could not articulate how the process of decentralising the HS unfolded was excluded from the study.

###  Study Participants 

 Participants in this study were DHMT members in Machinga and Central decision-makers at the ministries of health and local government headquarters. They also included directors and senior officers from the Office of the President and Cabinet (OPC) and the Department of Human Resource Management and Development. Table illustrates the characteristics of participants for the study.

**Table:** Characteristics of Participants

	**Education Qualification**	**Experience**	**No. of Central Decision-Makers**	**No. of DHMT Members**	**Total**
Male	At least first degree	10 years or more	8	11	19
Female	At lest first degree	10 years or more	2	4	6
Total			10	15	25

Abbreviation: DHMT, District Health Management Team.

 DHMT members were selected for the study because they are key actors with a variety of *macro* and *micro-level* activities depicting how decentralisation of the HS became a reality. Macro-level activities are generally implemented at the national level, while micro-level activities are implemented locally at the individual level. Although some macro-level activities (eg, planning) were initiated and implemented at the national level by central decision-makers, it is notable that some planning activities were also implemented at the district level by the Directorate of Planning and members of the DHMT. In a slightly different vein, micro-level activities (eg, service provision) were implemented locally at the individual level. Notably, members of the DHMT were responsible for local decision-making and coordination of health service provision at the local level before the roll-out of decentralisation. Furthermore, they were reporting to the Ministry of Health (MoH) Headquarters through the Regional Health Office. However, after decentralisation, they were reporting to the MoH Headquarters through the Health Zone Office. On the other hand, central decision-makers at the MoH Headquarters were responsible for policy formulation and international representation before and after decentralisation.

 Respondents from the OPC were included in the study because the OPC is the central oversight office for managing government reforms. Central decision-makers were selected because they are key policy-makers and prime movers in the process of decentralising the HS to the district. Some respondents were interviewed twice and a total of 36 individual in-depth and semi-structured interviews were conducted with 25 participants involved in this study.

###  Data Collection Method(s)

 The study involved identifying critical incidents from data using face-to-face in-depth and semi-structured interviews, and an interview guide was used as a tool for data collection. The face-to-face interview method was preferred for data collection because it allowed the researcher to probe participants in areas that needed clarity.

 To avoid missing important data, interviews were audio-recorded and transcribed immediately after each field visit as proposed by Strauss and Corbin^35^ because no one is certain about what pertains and what does not, so it is better to transcribe everything; otherwise, important data will be missed.

###  Data Analysis

 This study employed the Straussian data analysis, which is a multi-step process consisting of three stages: open coding, axial coding, and selective coding.^35^ The data analysis procedure for GT as proposed by Strauss and Corbin, is conducted concurrently with data collection. This entails that the analysis occurs iteratively with further data collection, followed by the resultant analysis, which guides the ongoing data collection. The coding process is aimed at deconstructing the data into convenient chunks that are intended to assist in understanding the phenomenon in question.^35^ Transcribed data were subjected to member checks and analysis using open coding and constant comparison. Member checking involved giving participants transcribed data of their respective interviews to ascertain the accuracy of information. Advocates of the GT methodology suggest that the emerging theory should be allowed to guide the researcher in identifying different portions of interview material that need to be transcribed for more detailed analysis.^35^ As such, it was important to first transcribe the early interviews and then code them to get a picture of emerging themes. Furthermore, some extracts from interviews were selected verbatim as quotations to illustrate specific issues because the wording captured the essence of concepts or meanings conveyed by the interviewees.

 As theprocess ofdata analysis continued, key categories were induced from data and were subsequently developed into dominant themes reflecting what central decision-makers were doing to decentralise the HS and what members of the DHMT were doing after decentralisation. Details of the research process and direct quotes from participants are given to serve as an audit trail and to enhance dependability and credibility. Further analysis was carried out using constant comparison, theoretical sensitivity, and theoretical sampling. At this stage, the initial core category emerged, which was about activities and processes of members of the DHMT and central decision-makers that frustrated change. As the researcher engaged in further theoretical sampling and data analysis, the final core category began to emerge, which provided more conceptual clarity in explaining the interviewees’ activities and processes.

## Results

 This study produced a GT which revealed that decentralisation of the HS to Machinga had mixed results with more predominant negative outcomes than positive effects due to inertia by some central and district-level actors during decentralisation. In presenting the findings, the focus will be on the seven different activities and processes, two opposing and interactional patterns of activities and processes, the theory of decentralisation derailed by inertia, and the consequences.

###  Seven Different Activities and Processes of Decentralising the Health System

 This study identified seven broad activities and processes of decentralising the HS by central decision-makers, and those practiced in a decentralised HS by members of the DHMT. More importantly, the first three broad categories of activities related to (1) *striving to decentralise the HS, (2) directing without alignment and commitment*, and (3) *enabling* governance and threshold capabilities, reflect how central decision-makers were implementing decentralisation between 2004 and 2022. The next four broad categories of activities and processes related to (4) *struggles to gain internal organisational efficiency *in a decentralised HS, (5) *collaboration for local health services* as decentralisation unfolded, (6)* perpetuation of a poor maintenance culture* in a decentralised HS, and (7) *impeding internal organisational effectiveness*. These illuminate how members of the DHMT largely implemented decentralised activities and processes after 2004. Based on the patterns which emerged during analysis, the above-mentioned seven activities and processes were categorised into two patterns: (1) *enabling pattern* supporting decentralisation, and (2) *impeding pattern* constraining decentralisation. These two patterns are presented below:

###  Enabling Pattern of Activities and Processes That Supported Decentralisation

 The study identified two activities and processes that supported the process of decentralisation and formed the enabling pattern as presented below:

####  Enabling Governance and Threshold Capabilities

 The factors labelled as* “enabling governance and threshold capabilities” *refer toavariety ofactivities and processes that were supportive of the process of decentralising the HS. They include: building threshold capabilities of human resources, cultivating a sense of collective accountability, and distributed health governance through structures. One DHMT member revealed a variety of governance structures that enhanced accountability as follows:

 “*Four structures were set up at the district headquarters after local elections in 2000. They include: (1) the district council, consisting of elected councillors, members of parliament, traditional leaders, and five representatives of special interest groups; (2) [the] district executive committee; (3) [the] health and environment committee [HEC]; and (4) [the] DHMT” *(DHMT member A).

 Members of the DHMT propounded that the establishment of governance structures in line with decentralisation was important because it enabled public officers to account for their actions in their manner of governing the provision of health services. This view was surmised by a member of DHMT (F) in this way:

 “*For some time, we have not been held accountable for our manner of governing the delivery of health services and responding to institutions from where we derive our authority. So far, with the coming of councillors, we have managed to establish governance structures. For example, we have [the] health and environment committee (HEC) and the hospital advisory committees (HAC) at the district headquarters that hold us accountable for our actions. When the district hospital runs out of medicines, we explain to the committees why the situation is like that and the action taken” *(DHMT member F).

####  Collaborating for Local Health Service Delivery

 The phenomenon of “*collaborating for local health service delivery”* reveals actions of DHMT members when operating in a decentralised HS in Machinga, Malawi. These activities involve (1) operational collaboration within the local health service provision; and (2) strategic alliance with external institutions outside the government system. The second set of activities reflects the internal forms of integrated service provision used in a resource-constrained organisational context. These included combining and delivering services at a point of delivery by one provider, and assigning the performance of multiple activities to one provider at a health facility on one field visit.

 Predominantly, the role of the district health office (DHO) was to coordinate service level agreement, which was a loose contract between the DHO and the Christian Health Association of Malawi (CHAM). The idea was to allow CHAM facilities provide some essential services to poor communities at a fee payable using donor funding. One member of the DHMT illuminated this as follows:

 “*The decision to sign a contract with CHAM facilities is really a good initiative. It has saved lives. Patients who have been denied access to health services in some areas now have access to the service for free. We coordinate and ensure that CHAM facilities deliver the required health services” *(DHMT member E).

 While the DHO’s office was the epi-centre of coordination and implementation, it was excluded from determining prices for each service provided under service level agreement at the point of delivery. This rendered the DHO’s coordination ineffective, and only served to expose resistance to decentralisation by central decision-makers. In a slightly different vein, collaboration was manifested by integrating service provision as expressed by one DHMT member in this way:

 “*We have been able to integrate service provision in our health facilities. With one service provider, we are able to provide several services at the point of delivery. For example, one provider has been able to take vital signs as well as mid-upper arm circumference (MUAC) for patients at the point of delivery. In so doing, we have temporarily managed to resolve the problem of inadequate funding and staff. The problem is that we can only combine a limited range of services when visiting a community due to people’s scope of expertise” *(DHMT member B).

###  Impeding Pattern of Activities and Processes That Constrained Decentralisation

 This study identified five activities and processes that constrained decentralisation of the HS to form an impeding pattern as follows:

####  Impeding Internal Organisational Effectiveness

 The factors labelled as “*impeding internal organisational effectiveness” *involved practices that frustrated decentralisation of the HS in the district. These include: (1) conflicting structural configuration, (2) organisational constraints nurtured by human resources, (3) bureaucratic resistance, and (4) corrupting service delivery. The study established that decentralisation was marred by conflicts emanating from dual administration where local healthcare managers had two reporting relationships. In this regard, the Director of Health and Social Services (DHSS) reported to two superiors, namely, the District Commissioner (DC) on administrative matters, and Secretary for Health on technical issues. Notably, the DHSS tended to follow instructions more from the Secretary for Health than the DC. This conflict slowed down the pace of implementing decentralisation, as accentuated by a member of the DHMT in this manner:

 “*The DHSS tends to take instructions more from the parent MoH than the DC. You know, one cannot serve two masters at a time… and cut the arm that feeds him…The DHSS leans towards the side that provides him with [his] daily bread and butter” *(DHMT member F).

 Another participant highlighted the nature of corruption in the district by revealing that it occurred in the form of kick-backs from suppliers in return for business. One DHMT member revealed how an officer responsible for procurement corruptly forced suppliers to pay kick-backs (bribes) in return for business as follows:

 “*One supplier approached me in 2019 to report that an officer responsible for procurement had been calling him at night demanding for a 10 per cent commission [kick-back]. He even told me that the said officer threatened him to pay the commission, or else his name would be scrapped-off from the list of suppliers” *(DHMT member E).

**Figure 1:**
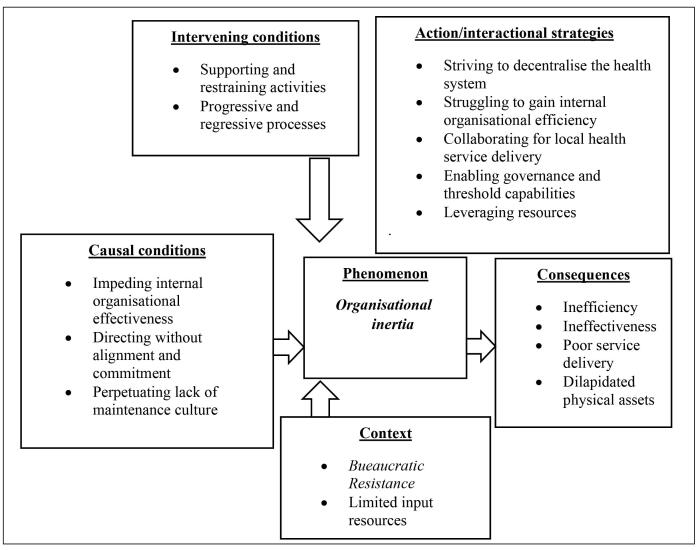
Initial and Final Core Categories of *Resistance* & *Organisational Inertia.*

 The findings of this study as per GT methodology indicate that while the enabling force was the basis for decentralisation, it is the force which impeded decentralisation that was powerful. Further analysis developed an initial core category of “*Bureaucratic resistance”* which translated to the final category of *“organisational inertia”* illustrated in Figure 1.

####  Striving to Decentralise the HS

 The phenomenon of *“striving to decentralise the HS”* involved a variety of activities and processes initiated by central decision-makers to drive decentralisation as a policy direction. These activities were implemented incrementary within a defined timeline and involved three stages: (1) initiating change, (2) implementing change, and (3) monitoring and modifying change processes. The director of planning (M) in the MoH recollected how the gradual, incremental, and phased approach to decentralisation was initially about devolving the planning function before devolving other functions, in a total of six different phases as depicted below.

 “*We agreed to devolve all functions and power to the district within a defined 10-year period. It was thought that this period was long enough to complete a planned change. We also adopted a gradual and phased approach to incremental decentralisation in order to monitor progress and allow for modifications, before the reform programme came to an end. The first phase of decentralisation was devolution of planning to the district. Our aim was to allow the DHMT to plan for themselves based on the needs of the district. The second phase was devolution of functions, for example, disciplining of staff. This was followed by phase three which focused on devolution of other recurrent transactions (ORT) budget. Phase four was devolution of human resources and phase five was designated to devolution of development budget. Finally, phase six was set aside for devolution of assets” *(Participant M).

 Although central decision-makers were devolving decision-making powers to the district, they harboured an inner psychological resistance to the decentralisation process. This exacerbated the continued struggle between policy implementation and resistance, ultimately dragging the scheduled power shift to the district. These sentiments were expressed by one member of the DHMT as follows:

 “*The Central Government is solely to blame. Senior officers are unwilling to shed-off some of their powers to the district. They are clinging onto power. It is difficult to tell when devolution will be completed” *(DHMT member A).

####  Directing Without Alignment and Commitment

 The phenomenon of* “directing without alignment and commitment”* involved activities of grappling with misalignment between resources available and decisional power, resisting effective human capability development, and restoring bureaucratic structures. These activities are exemplified by the imbalance between underfunding of the health sector and expanded responsibilities at the local council. The imbalance in financial resources and expanded responsibilities emanated from the central actors’ action of not increasing financial allocation to the district despite donor pull-out. One DHMT member was explicit about inadequate funding that stifled service delivery and the enormous responsibilities clearly being mismatched with few financial resources as follows:

 “*Decentralisation entails responsibilities transferred to the district should match with financial resources provided. Surprisingly, there is a discrepancy in the way funding allocation is made. Enormous responsibilities are matched with very few financial resources” *(DHMT member B).

 Clearly, there was an imbalance in financial resources allocated to the district and the expanded responsibilities arising from devolved functions. This imbalance reflected inertia by central actors that derailed decentralisation of the HS to the district.

####  Struggling to Gain Internal Organisational Efficiency 

 The phenomenon of *struggling to gain internal organisational efficiency *refers to activities of members of the DHMT when implementing the decentralisation policy. These activities include: upgrading security services to protect facility assets; contracting-out of non-core services to enhance efficiency gains; and recruitment to address the challenge of managing with inadequate junior staff. One DHMT member revealed how they struggled to gain internal organisational efficiency as follows:

 “*We experience shortage of data clerks, and their inadequacy leads to unavailable of data by the 10*^th^ *of every month. Sometimes we fail to enter data into the system because some programme coordinators are overwhelmed with work. This compromises decision-making*” (DHMT member I).

 Another DHMT Member focused on persistent theft of government property in public health facilities, and the ineffectiveness of security guards employed by the District Council. Below is a quote on how he expressed his concern:

 “*There were reported cases of theft of government property at the District Hospital, and [at] Mangamba, Nyambi, Machinga, Ntaja and Namanja health centres. Two water-tanks were stolen at Namanja and Nyambi health centres. Medicines, for example, paracetamol and amoxicillin, were also stolen at Ntaja Health centre. This theft of government property forced us to engage external independent security providers to strengthen security” *(DHMT member E).

####  Perpetuating Lack of Maintenance Culture

 The activity of *“perpetuating lack of maintenance culture”* captures the indifferent attitude and lack of determination by the DHMT and central actors to maintain physical assets in health facilities. This was manifested by the entrenched lackadaisical approach towards maintenance culture reflected in three ways: (1) indifference towards rehabilitating facility buildings, (2) nature of maintenance of medical equipment, and (3) type of servicing of office fleet.

 The study revealed that rehabilitation of facility buildings was the complex responsibility of central decision-makers, as the function was not yet devolved to the district. This is what one DHMT member emphasised about the effects of dilapidation during the rainy season and the general lackadaisical approach by the central government:

 “*All health facilities in the district have never been fully rehabilitated. The situation worsens during the rainy season because this hospital leaks a lot. Check the roof of this hospital… You will observe that timber supporting it is eaten up by ants and it may collapse any time. Sometimes we fear that the roof may collapse and injure patients” *(DHMT member E).

 The lackadaisical approach towards maintenance culture was not only reflected in failure to maintain buildings, but also in erratic preventive maintenance of medical equipment due to underfunding. This was revealed by a member of the DHMT (J) in the following quotation:

 “*Some of our medical equipment are not functioning properly because they are too old. Look, our sterilizer is in a state of disrepair. Of late, we have not accorded it the utmost priority it deserves. Although it is old, preventive maintenance could have helped to keep it in shape. We are now forced to go to either Zomba Central Hospital or Balaka or Ntcheu district hospitals to sterilise our equipment and gowns” *(DHMT member J).

###  Two Opposing and Interactional Sub-processes of the Activities

 In examining the actions of central decision-makers and members of the DHMT in Machinga, two interactional and opposing sub-processes of decentralisation of the HS were identified, namely: (1) *enabling governance and threshold capabilities*, and (2) *impeding internal organisational effectiveness*. While enabling factors supported change, impeding factors frustrated it. A central decision-maker surmised how policy-makers and members of the DHMT neglected inner psychological factors of individuals that derailed decentralisation as follows:

 “*We neglected inner psychological factors of individuals that are central to resisting change in our implementation of decentralisation. I believe that we focused much on external factors that either improve or impede change, for example, structural configuration of reporting relationships instead of inner emotional factors ( eg, fear of losing power)” *[Central Decision-maker N].

 The impeding pattern in this study has been referred to as *bureaucratic resistance,* and herein after called *organisational inertia*. The central feature of *organisational inertia* in this study is the dominance of resistance in various ways and at different stages of the decentralisation process. The above-mentioned two interactional and opposing sub-processes of decentralisation form the basis of the general theory of “*decentralisation derailed by organisational inertia.”*

###  Consequences: Derailment of Decentralisation

 As reiterated above, the central idea in the theory is *organisational inertia* manifested by resistance to change. It therefore follows that inertia derailed decentralisation, ultimately resulting in inefficiency, ineffectiveness, poor service delivery, and dilapidated assets. A member of the DHMT surmised how central decision-makers were in control but not actually ready to relinquish control over resources and budgeting to Machinga as shown below:

 “*The major challenge with decentralisation is bureaucratic resistance through unwillingness by central actors to hand-over functions and power to us. Until today, central actors develop the budget template for us and dictate the budget ceiling for use in planning. Resistance is also manifested by actions of district health actors who ask patients to pay for services that are meant for free. What is more disturbing is the fact that the Ministry of Local Government encourages other ministries to devolve their power and functions when the ministry itself is reluctant to shed-off some of its powers and functions” *(DHMT member B).

## Discussion

 The purpose of this study was to develop a substantive GT that elaborates on the transition of a HS to a decentralised model in Machinga, Malawi. This study developed a GT labelled *“decentralisation derailed by organisattional inertia,”* which suggests that decentralisation derailed due to resistance from the upper organisational echelons and members of the DHMT.

###  Model Depicting the Theory of Decentralisation Derailed by Organisational Inertia

 The substantive GT generated in this study is illuatrated by means of a model depicting seven activities and two interactional and opposing sub-processes of *enabling* and *impeding* patterns, which ultimately formed the overall process of decentralising the HS.

**Figure 2:**
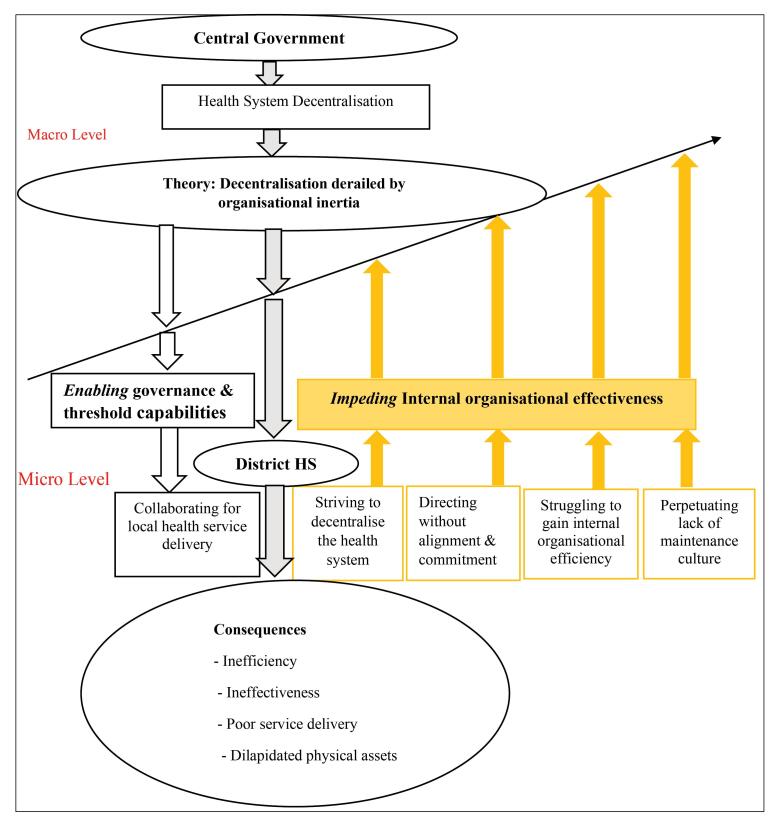
Model of the Theory of “Decentralisation Derailed by Organisational Inertia.” Source: Researcher’s own construction. Abbreviation: HS, health system.

 The theory elaborates that while *enabling* activities supported decentralisation, *impeding* actions were prevalent and dominant, thereby constraining the process of decentralising the HS at various levels of the HS. The model uses two sets of arrows to illustrate two opposing forces of the transition process. The upward arrows illustrate the opposing force, while the few and short downward arrows depict the enabling force, which is outweighed by the impeding one. The theory is explicit that the dominance of *impeding* activities resulted in negative consequences that derailed the process of decentralising the HS. These consequences include: inefficiency, ineffectiveness, poor service delivery, dilapidated physical assets, and increased burden of diseases. Figure 2 illustrates the model, which summarises the theory.

 As illustrated in Figure 2, the study revealed *seven* key elements that are linked to the consequences of decentralisation, and are oriented towards the derailment of decentralisation of the HS in the district. First, the GT illustrated by the model above suggests that members of the DHMT had a narrow decision-making power over resources and responsibilities compared to central decision-makers who had a wider decision-making power. This was exemplified by the central government’s determination to provide low funding to the district for service provision through a pre-determined budget ceiling around which the district could budget. Ideally, decentralisation entails a wider decision-making power by the DHMT at the local level to improve efficiency in service provision.^14^ The revelation for a narrow decision-making power by the DHMT over funding as established in this study supports the decision-space model by Bossert.^41^ The decision-space model is about the range of effective choices available to local managers (the DHMT), along a series of key decisional areas that are viewed as wide, moderate, and narrow.^14^

 It is notable that the decision-space theory has been used in case studies in many countries, for example, India,^10^ Philippines,^7,12^ Tanzania,^13^ and Ghana, Malawi, and Uganda.^14^ The majority of these studies examined the degree of authority that local decision-makers have over HS functions and found that managers at the local level had narrow decision-space which compromised service delivery. The above highlighted results are in line with findings of this study which suggest that in Malawi decentralisation derailed primarily due to the narrow-decision-making space by members of the DHMT in Machinga.

 Second, resistance by the upper organisational echelons at the strategic level of leadership and actions of some district healthcare managers actually contributed to the derailment of the decentralisation process in Malawi. The argument, in this case, is that forces against change were more pronounced and dominant over forces for change due to resistance. A similar study of reactions towards organisational change in Malaysia suggests that despite the need for change, many change initiatives fail because of resistance.^42^ This is because individuals think of change as a shock that immensely affects them negatively. Consistent with this result, Amarantou et al^43^ agree that resistance has been proven to be the main failure cause of all change initiatives across the globe because it derails service provision.

 Third, it is insightful to note that the derailment of decentralisation in this study was largely manifested by two forces of change labelled as *enabling and impeding *patterns. While the *enabling pattern *supported the change, the *impeding pattern* was prevalent and dominant, thereby frustrating the transition process at various levels of the HS. Thus, the central feature in this study was the dominance of the opposing force, which evolved into organisational inertia that derailed decentralisation. These twin iterative sub-processes of *enabling* and *impeding* patterns are like two sides of the same coin, constituting the overall process of *decentralisation derailed by organisational inertia.* A similar study was conducted by Pearse^44,45^ in South Africa where two patterns of leadership roles were identified that either enhanced or compromised the credibility of the leader and by implication, affected the success of the change process. The findings of this study are discussed from the perspective of the inertial theory, thereby contributing to understanding the role of inertia in building resistance within the context of decenralisation of the HS.

 Fourth, the findings of the study depict a multi-level transition process, which is complex, dynamic and unfolds over time. The multi-level perspective of decentralisation of the HS revealed that the transition process involved the central level actors relinquishing their powers and functions to the district-level actors. This involved the shift in the central role of providing services to the district mode of service provision. A similar study by Super et al^46^ showed that inter-governmental action between local managers and policy-makers at the headquarters evolves through congruent processes at different levels that changed institutional logics. The study indicated that multi-level perspective of transitions provides an in-depth understanding of the dynamics of evolving systems by seeing transition as a long-term process with co-evolving changes at multiple levels.

 Similarly, the study revealed complex reporting relationships where the DHSSs reported to two superiors at different levels instead of one. He reported to the Secretary for Health at the Headquarters through the Health Zone Office on technical matters, and to the DC in Machinga on administrative issues. This study posits that complex reporting relationships, as manifested by conflicting structural configurations, is in line with the literature on dual-administration, which contravenes the principle of unity of command. The principle of unity of command advocates that an employee should report to one and only one superior.^47^

 Fifth, decentralisation is a process, not an event or snapshot.^1,34,48^ Mindful that existing studies on decentralisation of the HS have predominantly focused on results, or outcomes,^22,49^ this study delved into the complex process of decentralising the HS to unravel the actions of central decision-makers and members of the DHMT in Malawi over time. Arguably, a focus on results or outcomes is fraught and simplistic as it strips decentralisation of its complexity. Given the above finding, this study is explicit that there is a compelling need for studies on decentralisation to focus on both process and outcomes rather than outcomes alone to capture complex processes of change over time.

 Sixth, Bracken^50^ is explicit that transition relates to intrinsic and psychological factors as people go through the process of change. It is notable that decentralisation of the HS in Malawi produced mixed results due to a focus on extrinsic factors (eg, structural rather than intrinsic factors). A focus on structural changes ignores the inner psychological dimensions of the transition process which translate to psychological inertia and ultimately overall organisational inertia. This study is explicit that psychological inertia at the personal and strategic levels of leadership entrusted with decentralisation of the HS contributed immensely to organisational inertia.

 Seventh, it is intriguing to note that inertial forces at different levels of the HS had a variety of consequences that derailed decentralisation in Malawi. Key among these forces is misalignment between local needs and the devolved responsibilities. Notably, resources made available for service provision at the local level could not match with devolved responsibilities. This finding is consistent with results of a study on decentralisation as a strategy for development in Ghana which suggests that decentralisation remained ineffective and inefficient because functions and responsibilities transferred to the local level were not accompanied by corresponding measures of resources.^51^ Primarily, under-resourcing of the HS in a decentralised set-up derails efficiency in service provision.

 Available literature in Malawi suggests that the HS has been underfunded over the years, in non-compliance with the Abuja Declaration, which encourages countries to allocate at least 15 percent of their national budget towards health.^52^ For example, it is reported that the central government allocated 9.3% of the total budget for the 2020/2021 fiscal year towards health,^24^ which was way below the portion stipulated in the Abuja declaration. Yoon et al^25^ observe that Malawi’s limited economic capacity restricted health expenditure to US$ 9.6 per capita in 2017, which fell well short of the World Health Organization’s (WHO’s) recommendation of US$ 86 per capita per annum needed for universal health coverage. This reluctance to relinquish power over resources and cede genuine autonomy to the district was at the core of inertia in the district that undermined policy implementation. Similar results are also reported in Ghana where partial decentralisation of resources was linked to misalignment of responsibilities.^3^

###  Limitations

 This study has two limitations. First, the use of one method of data collection, namely, interviews implied that the researcher relied on what members of the DHMT and central decision-makers at the headquarters chose to report during interviews. To address this limitation, the researcher employed the use of in-depth interviews to get depth on issues and also conducted follow-up interviews to seek clarification on what was earlier on reported by participants.

 The second limitation was the researcher’s dependence on recollections by district and central actors. Mindful that some of the critical incidents occurred a long time ago, there was a possibility that their recollections could not be perfectly accurate. To address this limitation, the study collected diverse incidents and compared them with those of various participants to saturate the data and understand the pattern of what happened over the years.

## Conclusion

 This article concludes that the psychological inertia at the personal and strategic levels of leadership entrusted with decentralising the HS in Malawi translated to organisational inertia, which contributed immensely to the derailment of shifting the HS from the centralised to the decentralised model of health service provision. In the decentralisation of the HS, the focus on structure and resources is critical but inadequate if transition of individuals is ignored and presents psychological inertia and resistance which leads to organisational inertia.

## Acknowledgement

 The authors acknowledge members of the DHMT and health practitioners in Machinga, and central decision-makers from the ministries of health and local government headquarters, OPC and department of human resource management and development for providing the data.

## Ethical issues

 This research study was approved by the Humanities and Social Sciences Research Ethics Committee of the University of KwaZulu-Natal, and the National Health Sciences research committee in Malawi.

## Competing interests

 Authors declare that they have no competing interests.

## Disclaimer

 The authors acknowledge that each has made a substantial contribution to the manuscript and are willing to take public responsibility or liability for any errors or omissions in this article.
